# Serotonergic Modulation of Nigrostriatal and Mesolimbic Dopamine and Motor/Exploratory Behaviors in the Rat

**DOI:** 10.3389/fnins.2021.682398

**Published:** 2021-08-12

**Authors:** Susanne Nikolaus, Hans-Jörg Wittsack, Christina Antke, Markus Beu, Hubertus Hautzel, Cvetana Decheva, Eduards Mamlins, Yuriko Mori, Joseph P. Huston, Gerald Antoch, Hans-Wilhelm Müller

**Affiliations:** ^1^Clinic of Nuclear Medicine, University Hospital Düsseldorf, Düsseldorf, Germany; ^2^Department of Diagnostic and Interventional Radiology, University Hospital Düsseldorf, Düsseldorf, Germany; ^3^Clinic for Nuclear Medicine, University Hospital Essen, Essen, Germany; ^4^Center for Behavioural Neuroscience, Institute of Experimental Psychology, Heinrich-Heine University, Düsseldorf, Germany

**Keywords:** D_2/3_ receptor, [^123^I]IBZM, 5-HT_2A_ receptor, altanserin, 2,5-dimethoxy-4-iodoamphetamine

## Abstract

**Purpose:** The 5-HT_2A_ receptor (R) is known to modulate dopamine (DA) release in the mammalian brain. Altanserin (ALT) and 2,5-dimethoxy-4-iodoamphetamine (DOI) act as 5-HT_2A_R antagonist and agonist, respectively. In the present study, we assessed the effects of ALT and DOI on motor and exploratory behaviors and on D_2/3_R binding in the rat brain with *in vivo* imaging methods.

**Methods:** D_2/3_R binding was determined after systemic application of ALT (10 mg/kg) or DOI (0.5 mg/kg) and the respective vehicles [dimethyl sulfoxide (DMSO) and 0.9% saline (SAL)] with [^123^I]IBZM as a single-photon emission computed tomography (SPECT) radioligand. Anatomical information for the delineation of the target regions was obtained with dedicated small animal MRI. Immediately after 5-HT_2A_R antagonistic or agonistic treatment, motor/exploratory behaviors were assessed for 45 (ALT) or 30 min (DOI) in an open field. Additional rats underwent behavioral measurements after injection of DMSO or SAL.

**Results:** ALT increased D_2/3_R binding in the ventral hippocampus relative to vehicle, while DOI augmented D_2/3_R binding in caudate putamen, frontal cortex, motor cortex, and ventral hippocampus. The 5-HT_2A_R agonist as well as antagonist decreased parameters of motor activity and active exploration. However, ALT, in contrast to DOI, decreased explorative head–shoulder motility and increased sitting.

**Conclusions:** The regional increases of D_2/3_R binding after ALT and DOI (90 and 75 min post-challenge) may be conceived to reflect decreases of synaptic DA. The reductions of motor/exploratory activities (min 1–45 and min 1–30 after challenge with ALT and DOI, respectively) contrast the regional reductions of D_2/3_R binding, as they indicate elevated DA levels at the time of behavioral measurements. It may be concluded that ALT and DOI modulate DA in the individual regions of the nigrostriatal and mesolimbocortical pathways differentially and in a time-dependent fashion.

## Introduction

Serotonin(5-HT)ergic innervation of the mammalian brain originates in the rostral and caudal raphe nuclei of the brainstem: the efferent projections of the rostal group ascend to the nuclei of the basal ganglia including the substantia nigra (SN; Wirtshafter et al., 1987), caudate putamen (CP; Steinbusch et al., 1980), globus pallidus (Eid and Parent, 2016), and subthalamic nucleus (Carpenter et al., 1981). Further projections rise to the ventral tegmental area (VTA; Oades and Halliday, 1987), nucleus accumbens (NAC; Ma and Han, 1992), thalamus (THAL; Moore et al., 1978), hypothalamus (Larsen et al., 1996), neocortex (Kievit and Kuypers, 1975), and limbic regions including the amygdala and hippocampus (HIPP; Köhler and Steinbusch, 1982), while others descend to the superior colliculus (Villar et al., 1988); the pedunculopontine tegmental, trigeminal, and cochlear brainstem nuclei (Li et al., 1993; Thompson et al., 1995; Steininger et al., 1997); the cerebellum (Torigoe et al., 1986) and the spinal cord (Kazakov et al., 1993). The caudal raphe nuclei project to the visceral and somatic motor nuclei, to the lateral reticular formation, and also to the spinal cord (Li et al., 1993; Manaker and Fogarty, 1995; Ribeiro-do-Valle, 1997).

In addition to 5-HT, the raphe nuclei contain a variety of other neurotransmitters including glutamate (GLU; Kaneko et al., 1990), γ-amino butyric acid (GABA; Belin et al., 1979), and dopamine (DA; Ochi and Shimizu, 1978). Thereby, the dorsal raphe nucleus sends DAergic efferents to NAC, CP, prefrontal cortex (PFC), and septum (Stratford and Wirtshafter, 1990). Neurochemical studies indicate that 5-HT modulates DAergic activity and DA release (for review, see, e.g., Alex and Pehek, 2007). Disturbances of both 5-HTergic and DAergic function have been implied in numerous psychiatric and neurological conditions (for review, see, e.g., Nikolaus et al., 2009a,b). Both neurotransmitter systems are relevant for a variety of functions including motor control, learning, and reward-seeking behavior (for review, see, e.g., Arias-Carrión and Poppel, 2007; Fischer and Ullsperger, 2017; Kawashima, 2018).

Altanserin (ALT) is a potent antagonist of 5-HT_2A_ receptors [R; dissociation constant (K_d_) = 0.3 nM; Kristiansen et al., 2005] with >100-fold selectivity over D_2/3_R, 5-HT_1A_R, 5-HT_6_R, and 5-HT_7_R (Leysen, 1989; Tan et al., 1999). 2,5-Dimethoxy-4-iodoamphetamine (DOI) has 5-HT_2A_R agonistic properties (K_d_ = 4 nM, Appel et al., 1990) with ~10-fold selectivity over 5-HT_2C_R (Canal et al., 2013).

In the only available behavioral study in rats, ALT [2.5 mg/kg subcutaneously (s.c.)] was observed to reduce locomotion (Kennett, 1992), while, in a microdialysis experiment (also in rats), intraperitoneal (i.p.) application of 20 mg/kg elicited an increase of striatal DA levels (Dewey et al., 1995).

In rats, DOI (0.25 mg/kg s.c., Krebs-Thomson et al., 1998; 0.3 and 1 mg/kg, s.c., Zaniewska et al., 2009; 0.25–4 mg/kg s.c., Hillegaart et al., 1996) reduced locomotion (Krebs-Thomson et al., 1998; Zaniewska et al., 2009) and rearing (Hillegaart et al., 1996; Krebs-Thomson et al., 1998). Decreases of rearing behavior were also observed by Hawkins et al. (2002) after intracerebroventricular (i.c.v.) and s.c. administration of 20–200 μg and 0.1–1 mg/kg, respectively. However, they found no effect on motor activity.

Systemic DOI (2 mg/kg i.p., Gudelsky et al., 1994; 2.5 mg/kg i.p. Ichikawa and Meltzer, 1995) exerted no effect on DA efflux in NAC (Ichikawa and Meltzer, 1995) and CP (Gudelsky et al., 1994; Ichikawa and Meltzer, 1995), whereas infusion into NAC (10–300 μM; Yan et al., 2000), CP (1 μM; Lucas and Spampinato, 2000), and PFC (300 μM; Bortolozzi et al., 2005) increased DA levels in these regions as well as in the VTA (Bortolozzi et al., 2005). Moreover, application of 0.1–10 mg/kg s.c. elevated DA release in the frontal cortex (FC; Gobert and Millan, 1999). These findings are in contrast to the report of Ng et al. (1999), who observed a decrease of DA efflux in the CP upon infusion of 10 and 20 μM of DOI into this region.

So far, little is known about the behavioral and neurochemical effects of ALT in rats. Moreover, findings on DOI have been controversial with respect to both neurochemistry and behavior. Also, *in vivo* imaging evidence is scarce: as of yet, one *in vivo* imaging study of striatal D_2/3_R binding has been conducted on baboons upon pretreatment with ALT [1 mg/kg intravenously (i.v.)], showing a reduction of [^11^C]raclopride binding in the CP indicative of an elevation of extracellular DA (Dewey et al., 1995). After treatment with DOI, so far, no *in vivo* imaging studies of D_2/3_R binding have been conducted on humans, non-human primates, or rats.

In the present study, we investigated the effects of systemic ALT (10 mg/kg i.p.) or DOI (0.5 mg/kg i.p.) on both motor/exploratory behaviors and D_2/3_R binding in regions of the rat nigrostriatal and mesolimbic systems, which are involved in motor as well as cognitive and emotional functioning [NAC, CP, THAL, SN/VTA, FC, motor cortex (MC), parietal cortex (PC), dorsal HIPP (dHIPP), ventral HIPP (vHIPP)], using small animal single-photon emission computed tomography (SPECT). Autoradiography studies have confirmed the presence of D_2/3_R binding sites for all of these areas, including those of the HIPP and neocortex (Bouthenet et al., 1987; Seeman and Grigoriadis, 1987; Morelli et al., 1990). Anatomical information for the delineation of the target regions was obtained with dedicated small animal MRI.

## Materials and Methods

### Animals

Studies were conducted on a total of 71 male Wistar rats (ZETT, Heinrich-Heine University, Düsseldorf, Germany), weighing 437 ± 51 g [mean ± standard deviation (SD); age, 3–4 months]. Rats were kept in standard Makrolon cages (590 × 380 × 200 mm; three animals per cage) in a climate cabinet (Scantainer, Scanbur BK, Karlslunde, Denmark; temperature, 20–22°C; air humidity, 60–70%) with an artificial light–dark cycle (lights on at 6:00 a.m., lights off at 6:00 p.m.). Temperature and air humidity were checked on a daily basis. Food and water were freely available. The protocol was approved by the regional authority (Landesamt für Natur, Umwelt und Verbraucherschutz, Nordrhein-Westfalen, Recklinghausen, Germany) and carried out in accordance with the European Communities Council Directive (86/609/EEC) and the German Law on the Protection of Animals.

### Study Design

**Figure 1:**
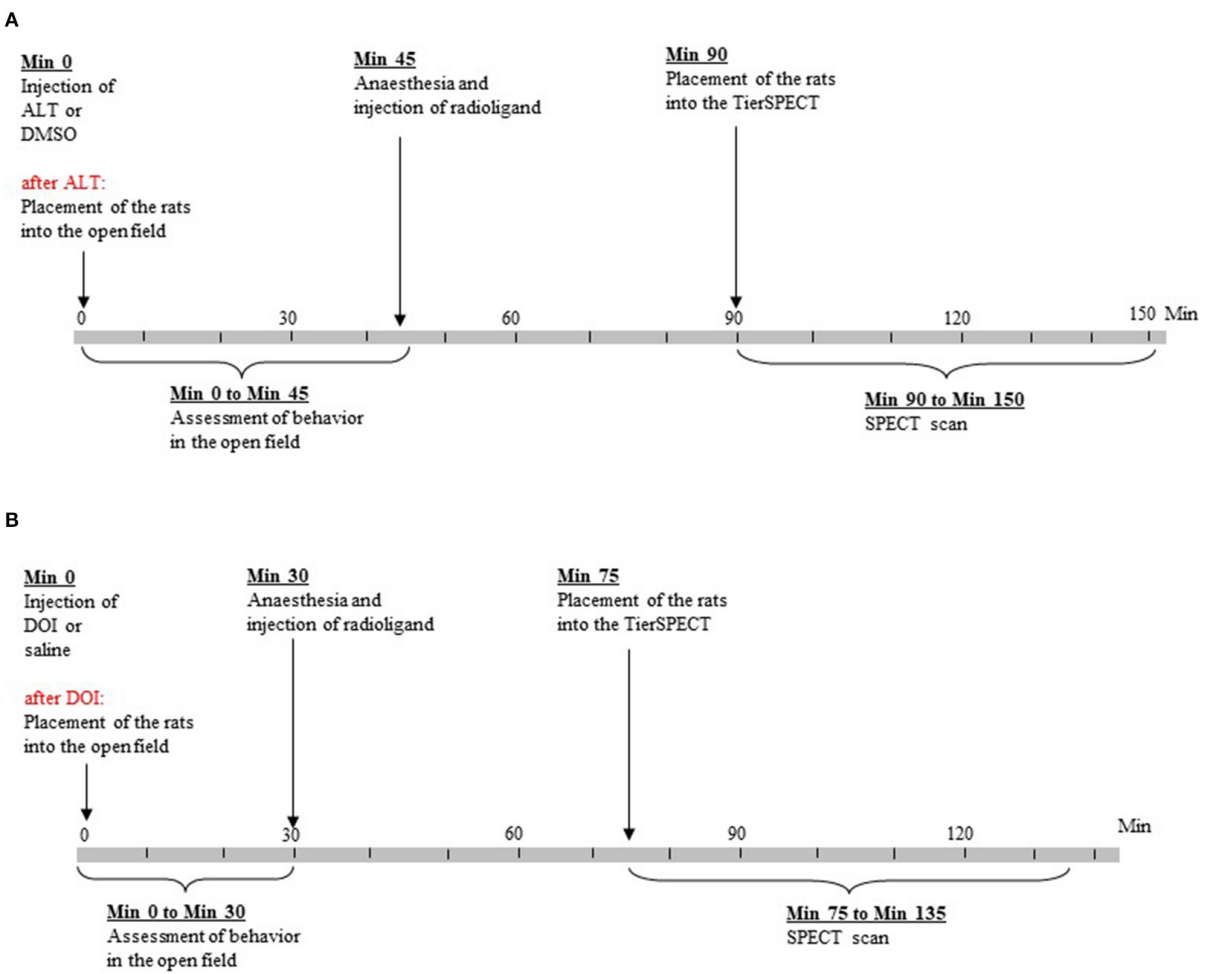
Time lines of experimental procedures. **(A)** Treatment with altanserin (ALT) and dimethyl sulfoxide (DMSO). **(B)** Treatment with 2,5-dimethoxy-4- iodoamphetamine (DOI) and saline.

Forty rats underwent (1) morphological MRI, (2) D_2/3_R imaging after 5-HTergic challenge (ALT or DOI), and (3) D_2/3_R imaging after administration of the respective vehicle [dimethyl sulfoxide (DMSO) or 0.9% physiological saline (SAL)]. Immediately after 5-HTergic challenges, rats underwent behavioral testing in an open field (Figure 1). The two SPECT measurements (including behavioral assessment after 5-HTergic challenges) were performed 7 days apart and in a randomized order. Due to cardiac arrest after the administration of the anesthetics, two rats merely underwent behavioral measurements (ALT, *n* = 1; DOI, *n* = 1) without subsequent D_2/3_R imaging.

In order to obtain comparative behavioral data, 31 further rats of the same strain, age, and weight merely underwent testing in the open field after precedent treatment with DMSO (*n* = 15) and SAL (*n* = 16).

### MRI Studies

After administration of ketamine hydrochloride (Ketavet®, Pharmacia GmbH, Erlangen, Germany; dose, 50 mg/kg i.p.; concentration, 100 mg/ml) and xylazine hydrochloride (Rompun®, Bayer, Leverkusen, Germany; dose, 2.5 mg/kg i.p.; concentration, 20 mg/ml), morphological imaging was performed with a dedicated small animal MRI (MRS3000 Pre-clinical MRI, 3.0 T, MR Solutions, Guildford, UK; coil diameter, 54 mm; field of view, 64 × 64 × 44 mm; spatial resolution, 0.25 × 0.25 × 0.69). High-resolution images were obtained by performing 3D fast low-angle shot (FLASH) sequences (image matrix, 192 × 192 × 96; echo time, 4.87 ms; repetition time, 30 ms; excitation flip angle, 30°; total acquisition time, 9.22 min; Haase et al., 1986).

### Drug Treatment

Rats received either ALT (Sigma-Aldrich, Taufkirchen, Germany; molecular weight, 411.49 g/mol; dose, 10 mg/kg i.p.; injection volume, 0.5 ml/kg; concentration, 20 mg/ml; *n* = 21), (±)DOI hydrochloride (Sigma-Aldrich, Taufkirchen, Germany; molecular weight, 357.62 g/mol; dose, 0.5 mg/kg i.p.; injection volume, 1 ml/kg; concentration, 0.5 mg/ml; *n* = 19) or the vehicles DMSO (for ALT; 100%; Sigma-Aldrich, Taufkirchen, Germany; molecular weight, 78.13 g/mol; dose, 0.5 ml/kg i.p.) and isotonic SAL (for DOI; B. Braun Melsungen AG, Melsungen, Germany; molecular weight: 58.5 g/mol; dose, 1 ml/kg i.p.).

In the available studies, ALT was behaviorally and pharmacologically active after systemic doses of 2.5 mg/kg (Kennett, 1992) and 20 mg/kg (Dewey et al., 1995), respectively. DOI proved behaviorally active after systemically applied doses between 0.05 and 4 mg/kg (Hillegaart et al., 1996), while neurochemical effects were observed between 0.1 and 10 mg/kg (Gobert and Millan, 1999). Based on these findings, we chose intermediate doses of 10 mg/kg of ALT and 0.5 mg/kg of DOI, respectively.

### Single-Photon Emission Computed Tomography Studies

The employed small animal tomograph (“TierSPECT”; Central Institute for Electronics, Research Center Jülich, Jülich, Germany) was described in detail elsewhere (Schramm et al., 2000). Briefly, the detector consists of a NaI(Tl) disk (thickness, 3 mm) coupled to a position-sensitive photomultiplier (Hamamatsu R3292) and mounted on a rotating gantry {field of view, 82 mm; tomographic resolution [full width at half maximum (FWHM)] for ^123^I, 3.4 mm; sensitivity for ^123^I, 16 cps/MBq}. In the present study, a low-energy ultra-high-resolution parallel-hole collimator (LEUHR, 37 × 1 × 0.2 mm^3^) was employed. Imaging data were recorded in a step-and-shoot mode over a circular orbit (radius of rotation, 65 mm) in angular steps of 6° (60 projections, 60 s/projection). The 15% energy window was centered on the 159-keV gamma photopeak of ^123^I. Data were acquired in a 128 × 128 matrix with a pixel width and a slice thickness of ≈0.664 mm, respectively. Reconstruction was performed with an iterative ordered-subset-expectation-maximization algorithm (three iterations, four subsets/iteration). No post-filtering procedure was applied. An attenuation correction was implemented assuming a uniformly attenuating medium (linear attenuation coefficient, 0.10 cm^−1^).

Upon anesthesia with ketamine hydrochloride (dose, 100 mg/kg i.p.; concentration, 100 mg/ml) and xylazine hydrochloride (dose, 5 mg/kg i.p.; concentration, 20 mg/ml), [^123^I]S-3-iodo-*N*-(1-ethyl-2-pyrrolidinyl)methyl-2-hydroxy-6-methoxy benzamide ([^123^I]IBZM; GE Healthcare, Munich, Germany; activity, 29.9 ± 3.4 MBq; concentration, 3.4 × 10^−9^ g/ml; specific activity, >74 TBq/mmol at reference time) was injected into the tail vein. With an applied mean radioactivity of 30 MBq, a mean animal weight of 437 g, a specific activity of >74 TBq/mmol, and an affinity of ~0.3 nM (Verhoeff et al., 1991), according to Hume et al. (1998), a D_2/3_R occupancy in the range of 2% may be expected.

In previous studies conducted with the “TierSPECT,” we have demonstrated the displaceability of this radioligand from the D_2/3_R binding site by endogenous DA (e.g., Nikolaus et al., 2016). In both humans (Verhoeff et al., 1991) and rodents (Verhoeff et al., 1991; Jongen et al., 2008), under various anesthetics including ketamine (Jongen et al., 2008).

Specific binding of [^123^I]IBZM in the striatum reaches its maximum at 40 min post-injection and remains stable for up to 2 h. This coincides with the time of maximum DA concentrations after administration of ALT (40–60 min post-challenge; Dewey et al., 1995) and DOI (20–60 min post-challenge; Gobert and Millan, 1999). In order to account for these time courses, data acquisition was started 45 min after radioligand administration (ALT and DMSO, 90 min post-treatment; DOI and SAL, 75 min post-treatment) and ended 60 min later (ALT and DMSO, 150 min post-treatment; DOI and SAL, 135 min post-treatment). Animals were kept under anesthesia from 5 min before radioligand application until the end of data acquisition, receiving total quantities of up to 60 mg of ketamine hydrochloride and 12 mg of xylazine hydrochloride.

### Behavioral Studies

Immediately after administration of ALT, DOI, DMSO, or SAL, motor and exploratory behaviors were assessed in an open field (Phenotyper®, Noldus Information Technology, Wageningen, The Netherlands; dimensions, 45 × 45 × 56 cm; illumination, 19 lx) with EthoVision XT (Noldus Information Technology, Wageningen, The Netherlands).

In previous microdialysis studies, DA peaks were reached at about 40 min (ALT; Dewey et al., 1995) and 20 min (DOI; Gobert and Millan, 1999) post-injection. Hence, for a total of 45 (ALT and DMSO) or 30 min (DOI and SAL; Figure 1), durations (s) and frequencies (counts) of the following behaviors were rated in blocks of 5 min by one of the investigators (SN): (a) ambulation (as measure of motor activity); (b) sitting (as measure of “passive immobility”; Müller et al., 2004); (c) rearing (as measure of active exploration); and (d) explorative movements of head, neck, and shoulders, while the animal was sitting. Thereby, head and shoulder movements related to grooming behavior were explicitly excluded. Furthermore, based on the movement of the animal's center point, EthoVision XT automatically determined the distance in cm covered by the rat. Since the center point of a rat shifts not only on account of horizontal movements but also on account of vertical and diagonal movements, as they occur in rearing behavior and head–shoulder motility, the resulting distances can be considered as a measure of overall motor activity. Behavioral studies were conducted between 9:00 a.m. and 5:00 p.m. Following the behavioral tests, rats were anesthetized as described above and injected [^123^I]IBZM.

### Evaluation of Single-Photon Emission Computed Tomography Imaging Studies

D_2/3_R imaging data were analyzed with PMOD (version 3.5, PMOD Technologies Ltd., Zürich, Switzerland). For each rat, SPECT and MR images were coregistered. Then, the MRI was coregistered with the Paxinos standard rat brain MRI (Schiffer et al., 2006) provided by PMOD. The necessary mathematical transformations were saved. The SPECT image as coregistered with the MRI was imported using these transformations, which allowed creation of an overlay with the Paxinos standard rat brain MRI. On these overlays, the following volumes of interest (VOIs) were defined: NAC, CP, THAL, SN/VTA, FC, MC, PC, dHIPP, and vHIPP. According to the rat brain atlas (Paxinos and Watson, 2014), all these regions have maximum craniocaudal and one-sided mediolateral and dorsoventral (vertical or oblique) dimensions in the range of or beyond the spatial resolution of the imaging system (3.4 mm for ^123^I; Schramm et al., 2000).

Regional binding potentials (BPs) were calculated according to the simplified reference tissue model (Ichise et al., 2001) by computing ratios of the radioactivity counts measured in the specifically bound compartments (NAC, CP, THAL, SN/VTA, FC, MC, PC, dHIPP, and vHIPP) to the radioactivity counts in the cerebellar reference VOI. Although the cerebellum receives 5-HTergic afferents from raphe nuclei (Torigoe et al., 1986), it is suitable as a reference region, since it contains practically no D_2/3_R binding sites (Camps et al., 1989).

### Statistical Analysis

#### D_2/3_R Imaging Studies

Distributions of regional BPs after 5-HTergic challenges as well as vehicles were tested for normality with the non-parametric Kolmogorov–Smirnov test (α ≤ 0.05). The regional BPs were not uniformly normally distributed after ALT or DMSO (0.001 ≤ *p* ≤ 0.200) nor after DOI or SAL (0.008 ≤ *p* ≤ 0.200).

Medians and interquartile ranges (25th/75th and 5th/95th percentiles) of regional BPs were calculated for each treatment. Moreover, percentual differences of BPs after ALT and DOI relative to DMSO and SAL, respectively, were computed.

A two-way analysis of variance (ANOVA) was conducted with the factors “brain region” and “treatment” (α ≤ 0.05). Regional BPs were compared between 5-HTergic challenge and the respective vehicle (ALT vs. DMSO, and DOI vs. SAL) with the non-parametric Wilcoxon signed-rank test for paired samples (two-tailed, α ≤ 0.05).

Statistic calculations were performed with SigmaStat (version 3.5, Systat Software Inc., Erkrath, Germany).

#### Network Analyses

With this mode of analysis, the network structure of variables—predefined so-called “nodes”—can be analyzed by estimating path coefficients, which describe the “strength” of the individual connections. Here, we separately assessed the associations between D_2/3_R binding in NAC, CP, THAL, SN/VTA, FC, MC, PC, dHIPP, and vHIPP after 5-HTergeic challenges and the respective vehicles. Covariance matrices were estimated with gaussian graphical models employing graphical L_1_ (lasso) regularized regression in order to increase matrix sparsity (Friedman et al., 2008). The tuning parameter was chosen using the extended Bayesian information criterion (EBICglasso). EBICglassos were computed using JASP (version 0.10.2.0, © 2013–2019 University of Amsterdam).

#### Behavioral Studies

For each pretreatment condition (ALT, DOI, DMSO, and SAL), distributions of behavioral parameters (duration and frequencies of ambulation, sitting, rearing, and head–shoulder motility) after 5-HTergic challenges as well as vehicles were tested for normality with the non-parametric Kolmogorov–Smirnov test (α ≤ 0.05). Since none of the behavioral parameters was uniformly normally distributed in any of the pretreatment conditions (0.0001 ≤ *p* ≤ 0.2), behaviors in each 5-min bin were compared between 5-HTergic challenge and treatment with the respective vehicle (ALT vs. DMSO, and DOI vs. SAL) with the Mann–Whitney *U*-test for unrelated samples (two-sided, α ≤ 0.05). Statistic calculations were performed with SigmaStat (version 3.5, Systat Software Inc., Erkrath, Germany).

## Results

### D_2/3_R Binding

#### 5-HTergic Treatments vs. Vehicle

**Figure 2:**
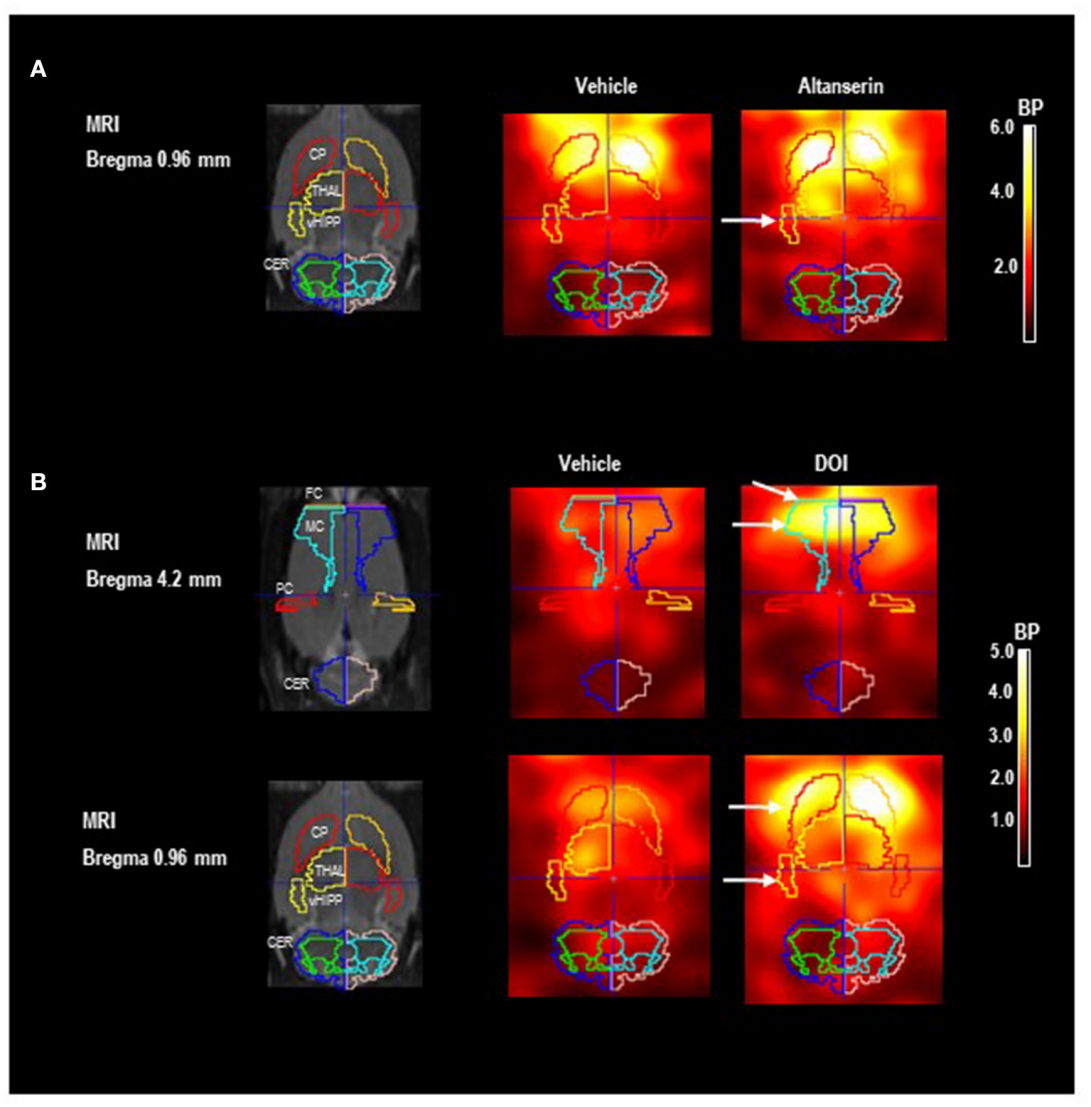
Paxinos standard rat brain MRI and D_2/3_R single-photon emission computed tomography (SPECT) images after 5-HT_2A_ receptor antagonistic **(A)** and agonistic treatment **(B)** with [^123^I]IBZM as a radioligand. The left column shows Paxinos standard rat brain MR images (Schiffer et al., 2006) at different positions from bregma together with the standard volume of interest (VOI) templates provided by PMOD. The middle column shows coronal SPECT slices in two characteristic rats after pretreatment with **(A)** dimethyl sulfoxide (DMSO) and **(B)** saline as vehicle at the same positions from bregma depicted in the left column. The right column shows SPECT slices in the same rats after pretreatment with **(A)** 10 mg/kg of altanserin and **(B)** 0.5 mg/kg of 2,5-dimethoxy-4-iodoamphetamine (DOI) at the same positions from bregma. Increases of [^123^I]IBZM accumulation are marked by white arrows. SPECT images show binding potentials (BPs). It is understood that the calculation of BPs is only valid for regions with specific radioligand binding. Analysis and image algebra were performed with PMOD (version 3.5, PMOD Technologies Ltd., Zürich, Switzerland). CER, cerebellum; CP, caudate putamen; FC, frontal cortex; MC, motor cortex; PC, parietal cortex; THAL, thalamus; vHIPP, ventral hippocampus.

Figures 2A,B show images of the Paxinos standard rat brain MRI atlas (Schiffer et al., 2006) at different positions from the bregma together with the standard VOI templates provided by PMOD (left column). The next two columns show characteristic images of regional [^123^I]IBZM accumulations on coronal slices after treatment with vehicle [DMSO (A) or SAL (B); middle] and after 5-HTergic challenge [ALT (A) or DOI (B); right], at the positions from the bregma depicted in the left column. Scans after vehicle and the respective 5-HTergic challenge stem from the same rat.

The two-way ANOVA yielded significant effects of “treatment” (*p* < 0.0001) and “brain region” (*p* < 0.001) as well as a significant interaction “treatment × region” (*p* = 0.024).

**Figure 3:**
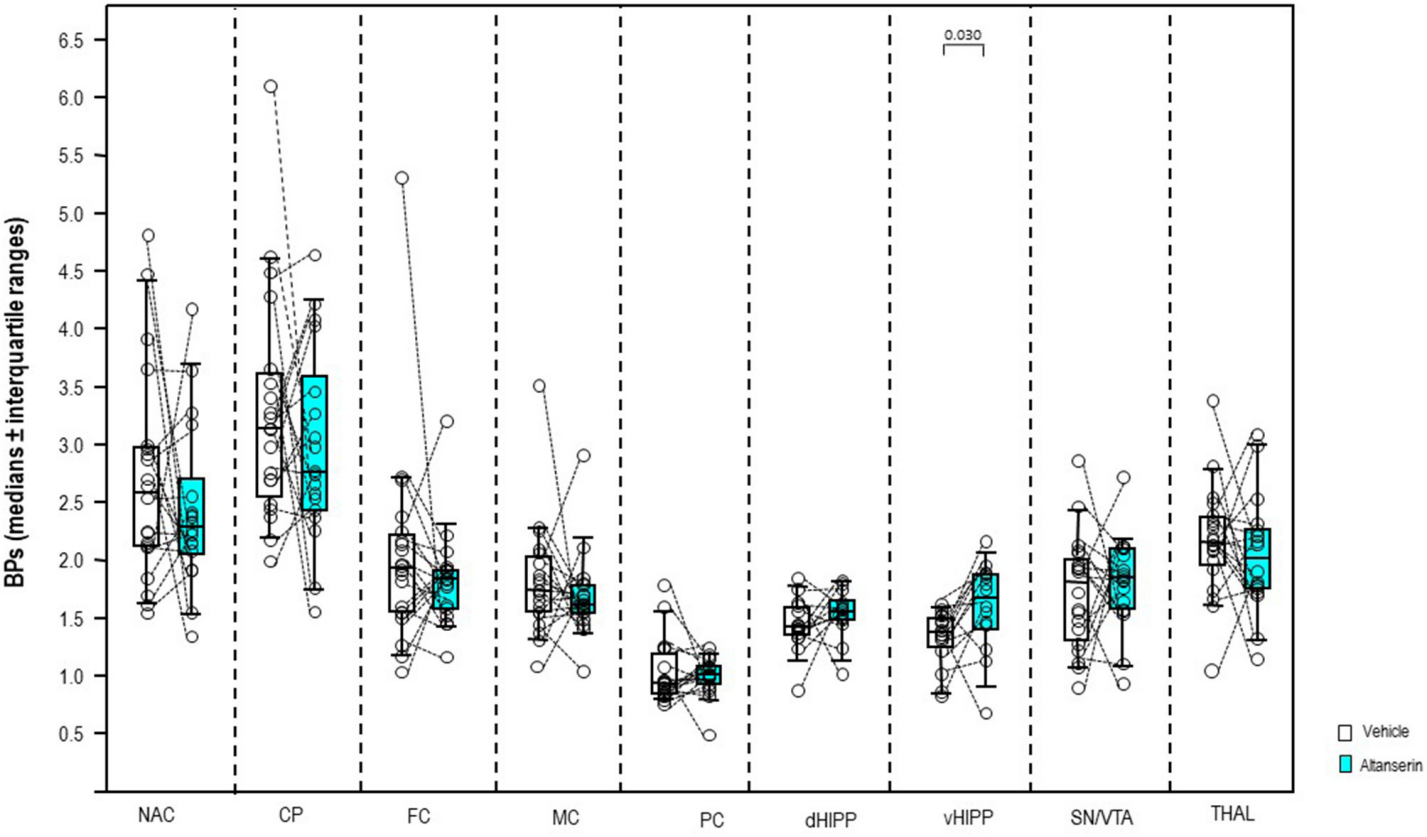
Binding potentials (BPs) after altanserin (10 mg/kg; turquoise) and vehicle (dimethyl sulfoxide; white). Rendered are medians and 25th/75th (boxes) and 9th/95th quartiles (whiskers). The circles represent the individual animals. Treatments were compared with the Wilcoxon signed-rank test for paired samples (two-tailed, α = 0.05). The significant *p*-values are given. CP, caudate putamen; dHIPP, dorsal hippocampus; FC, frontal cortex; MC, motor cortex; NAC, nucleus accumbens; PC, parietal cortex; SN/VTA, substantia nigra/ventral tegmental area; THAL, thalamus (THAL); vHIPP, ventral hippocampus.

After 10 mg/kg of ALT (Figure 3), BP was significantly augmented in vHIPP (+22%, *p* = 0.030) relative to vehicle. No significant alterations were observed in the other brain regions (0.268 ≤ *p* ≤ 0.899).

**Figure 4:**
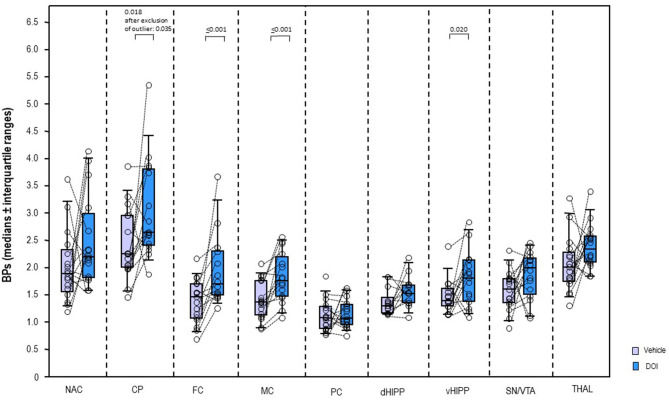
Binding potentials (BPs) after 2,5-dimethoxy-4-iodoamphetamine (DOI; 0.5 mg/kg; blue) and vehicle (saline; light purple). Rendered are medians and 25th/75th (boxes) and 9th/95th quartiles (whiskers). The circles represent the individual animals. Treatments were compared with the Wilcoxon signed-rank test for paired samples (two-tailed, α = 0.05). The significant *p*-values are given. CP, caudate putamen; dHIPP, dorsal hippocampus; FC, frontal cortex; MC, motor cortex; NAC, nucleus accumbens; PC, parietal cortex; SN/VTA, substantia nigra/ventral tegmental area; THAL, thalamus (THAL); vHIPP, ventral hippocampus.

In a dose of 0.5 mg/kg, DOI (Figure 4) induced significant increases of the BPs in CP (+17%, *p* = 0.018; after exclusion of the outlier: *p* = 0.035), FC (+16%, *p* ≤ 0.001), MC (+29%, *p* ≤ 0.001), and vHIPP (+30%, *p* = 0.020). No significant alterations were observed in the other brain regions (0.107 ≤ *p* ≤ 0.847).

#### Network Analyses

**Figure 5:**
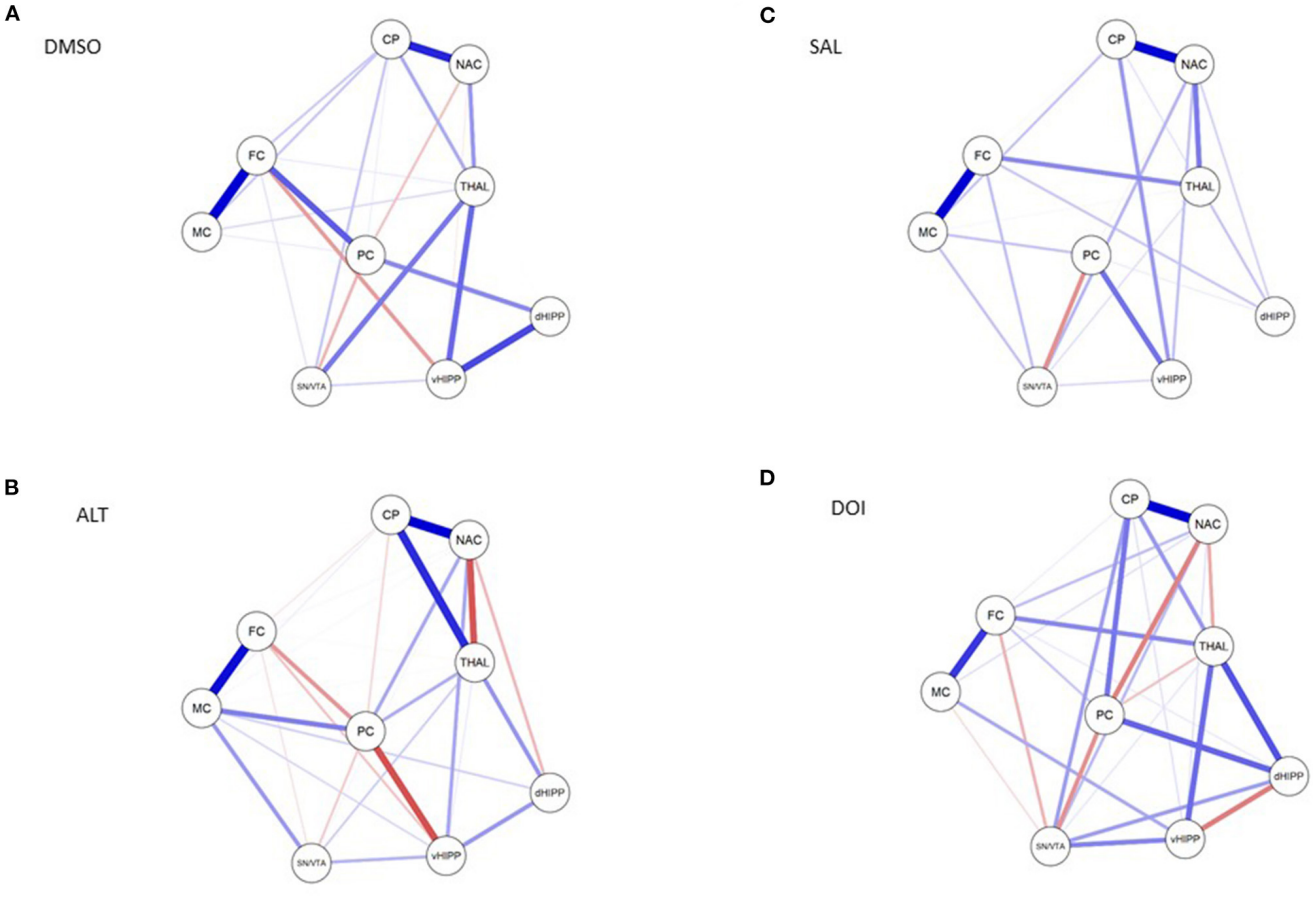
Connections between nucleus accumbens (NAC), caudate putamen (CP), thalamus (THAL), substantia nigra/ventral tegmental area (SN/VTA), frontal cortex (FC), motor cortex (MC), parietal cortex (PC), dorsal hippocampus (dHIPP), and ventral hippocampus (vHIPP) obtained after **(A)** dimethyl sulfoxide (DMSO), **(B)** altanserin (ALT), **(C)** saline (SAL), and **(D)** 2,5-dimethoxy-4-iodoamphetamine (DOI) application. Positive and negative associations are represented by blue and red lines, respectively. The size of the lines indicates the strength of the individual connections. Models were estimated with JASP (version 0.10.2.0, © 2013–2019 University of Amsterdam).

Network analyses of the BPs obtained after treatment with DMSO yielded 23 out of 36 possible connections (Figure 5A; sparsity, 0.361), while after treatment with ALT, 30 out of 36 possible connections were obtained (Figure 5B), decreasing sparsity to 0.167. The individual path coefficients (c) obtained after DMSO and ALT are given in Tables 1, 2, respectively. Common to both treatments were strong positive connections (c > 0.100) between NAC and CP, FC and MC, dHIPP and vHIPP, CP and THAL, SN/VTA and THAL, and SN/VTA and vHIPP, as well as negative connections between FC and vHIPP, and SN/VTA and PC. Moreover, connections between NAC and THAL and FC and PC were positive after DMSO but negative after ALT. Conversely, the connection between NAC and PC was negative after DMSO, but positive after ALT. Besides, the positive connections between CP and SN/VTA, CP and FC, CP and MC, and THAL and vHIPP as well as PC and dHIPP obtained after DMSO were not existent any more after treatment with ALT. Instead, positive connections between NAC and vHIPP, SN/VTA and MC, THAL and PC, THAL and dHIPP, MC and PC, MC and dHIPP, and MC and vHIPP as well as negative connections between NAC and dHIPP and PC and vHIPP were existent.

**Table 1:** Path coefficient matrix after treatment with dimethyl sulfoxide (DMSO) obtained with EBICglasso modeling of D_2/3_R binding potentials in nucleus accumbens (NAC), caudate putamen (CP), thalamus (THAL), substantia nigra/ventral tegmental area (SN/VTA), frontal cortex (FC), motor cortex (MC), parietal cortex (PC), dorsal hippocampus (dHIPP), and ventral hippocampus (vHIPP).

	**NAC**	**CP**	**THAL**	**SN/VTA**	**FC**	**MC**	**PC**	**dHIPP**	**vHIPP**
NAC	0.000	0.578	0.268	0.000	0.000	0.000	−0.148	0.000	−0.053
CP		0.000	0.218	0.148	0.134	0.142	0.043	0.000	0.000
THAL			0.000	0.350	0.056	0.093	0.000	0.004	0.395
SN/VTA				0.000	0.073	0.000	−0.187	0.000	0.114
FC					0.000	0.679	0.427	0.000	−0.253
MC						0.000	0.051	0.000	0.000
PC							0.000	0.306	0.000
dHIPP								0.000	0.481
vHIPP									0.000

**Table 2:** Path coefficient matrix after treatment with altanserin obtained with EBICglasso modeling of D_2/3_R binding potentials in nucleus accumbens (NAC), caudate putamen (CP), thalamus (THAL), substantia nigra/ventral tegmental area (SN/VTA), frontal cortex (FC), motor cortex (MC), parietal cortex (PC), dorsal hippocampus (dHIPP), and ventral hippocampus (vHIPP).

	**NAC**	**CP**	**THAL**	**SN/VTA**	**FC**	**MC**	**PC**	**dHIPP**	**vHIPP**
NAC	0.000	0.866	−0.567	0.033	0.030	0.028	0.287	−0.230	0.292
CP		0.000	0.713	0.000	−0.082	0.057	−0.133	0.000	0.000
THAL			0.000	0.155	−0.025	0.018	0.264	0.350	0.069
SN/VTA				0.000	−0.094	0.337	−0.166	0.000	0.248
FC					0.000	0.822	−0.342	0.000	−0.587
MC						0.000	0.427	0.153	0.124
PC							0.000	0.000	−0.587
dHIPP								0.000	0.344
vHIPP									0.000

Network analyses of the BPs obtained after treatment with SAL yielded 23 out of 36 possible connections (Figure 5C; sparsity, 0.361), while after treatment with DOI (Figure 5D), 29 out of 36 possible connections were obtained (Figure 5B), decreasing sparsity to 0.194. The individual path coefficients obtained after SAL and DOI are given in Tables 3, 4, respectively. Common to both treatments were strong positive connections (c > 0.100) between NAC and CP, NAC and SN/VTA, SN/VTA and vHIPP, THAL and FC, and THAL and dHPP as well as FC and MC and a strong negative association between SN/VTA and PC. After SAL, strong positive connections were obtained between NAC and THAL, NAC and PC, SN/VTA and FC, and SN/VTA and MC, which became negative after treatment with DOI. Moreover, after SAL, strong positive connections were found between NAC and dHIPP, NAC and vHIPP, CP and MC, CP and vHIPP, FC and dHIPP, MC and PC, and PC and vHIPP, which were not existent or much weaker after DOI. In turn, strong positive connections between NAC and FC, CP and SN/VTA, CP and THAL, CP and PC, CP and dHIPP, SN/VTA and dHIPP, THAL and vHIPP, PC and dHIPP, FC and PC, and MC and vHIPP and strong negative connections between THAL and PC as well as dHIPP and vHIPP were added.

**Table 3:** Path coefficient matrix after treatment with saline obtained with EBICglasso modeling of D_2/3_R binding potentials in nucleus accumbens (NAC), caudate putamen (CP), thalamus (THAL), substantia nigra/ventral tegmental area (SN/VTA), frontal cortex (FC), motor cortex (MC), parietal cortex (PC), dorsal hippocampus (dHIPP), and ventral hippocampus (vHIPP).

	**NAC**	**CP**	**THAL**	**SN/VTA**	**FC**	**MC**	**PC**	**dHIPP**	**vHIPP**
NAC	0.000	0.592	0.307	0.169	0.000	0.000	−0.033	0.103	0.145
CP		0.000	0.065	0.000	0.016	0.132	0.000	0.000	0.234
THAL			0.000	0.071	0.275	0.022	0.000	0.132	0.000
SN/VTA				0.000	0.146	0.138	−0.255	0.000	0.089
FC					0.000	0.604	0.000	0.126	0.000
MC						0.000	0.138	0.000	0.000
PC							0.000	0.045	0.327
dHIPP								0.000	0.000
vHIPP									0.000

**Table 4:** Path coefficient matrix after treatment with 2,5-dimethoxy-4-iodoamphetamine (DOI) obtained with EBICglasso modeling of D_2/3_R binding potentials in nucleus accumbens (NAC), caudate putamen (CP), thalamus (THAL), substantia nigra/ventral tegmental area (SN/VTA), frontal cortex (FC), motor cortex (MC), parietal cortex (PC), dorsal hippocampus (dHIPP), and ventral hippocampus (vHIPP).

	**NAC**	**CP**	**THAL**	**SN/VTA**	**FC**	**MC**	**PC**	**dHIPP**	**vHIPP**
NAC	0.000	0.648	−0.209	0.168	0.157	0.077	−0.310	0.000	0.047
CP		0.000	0.254	0.242	0.044	0.000	0.341	0.134	0.073
THAL			0.000	0.060	0.298	0.000	−0.144	0.433	0.368
SN/VTA				0.000	−0.183	−0.084	−0.272	0.240	0.304
FC					0.000	0.503	0.134	0.055	0.000
MC						0.000	0.000	0.000	0.074
PC							0.000	0.390	0.000
dHIPP								0.000	−0.321
vHIPP									0.000

### Motor and Exploratory Behaviors

**Figure 6:**
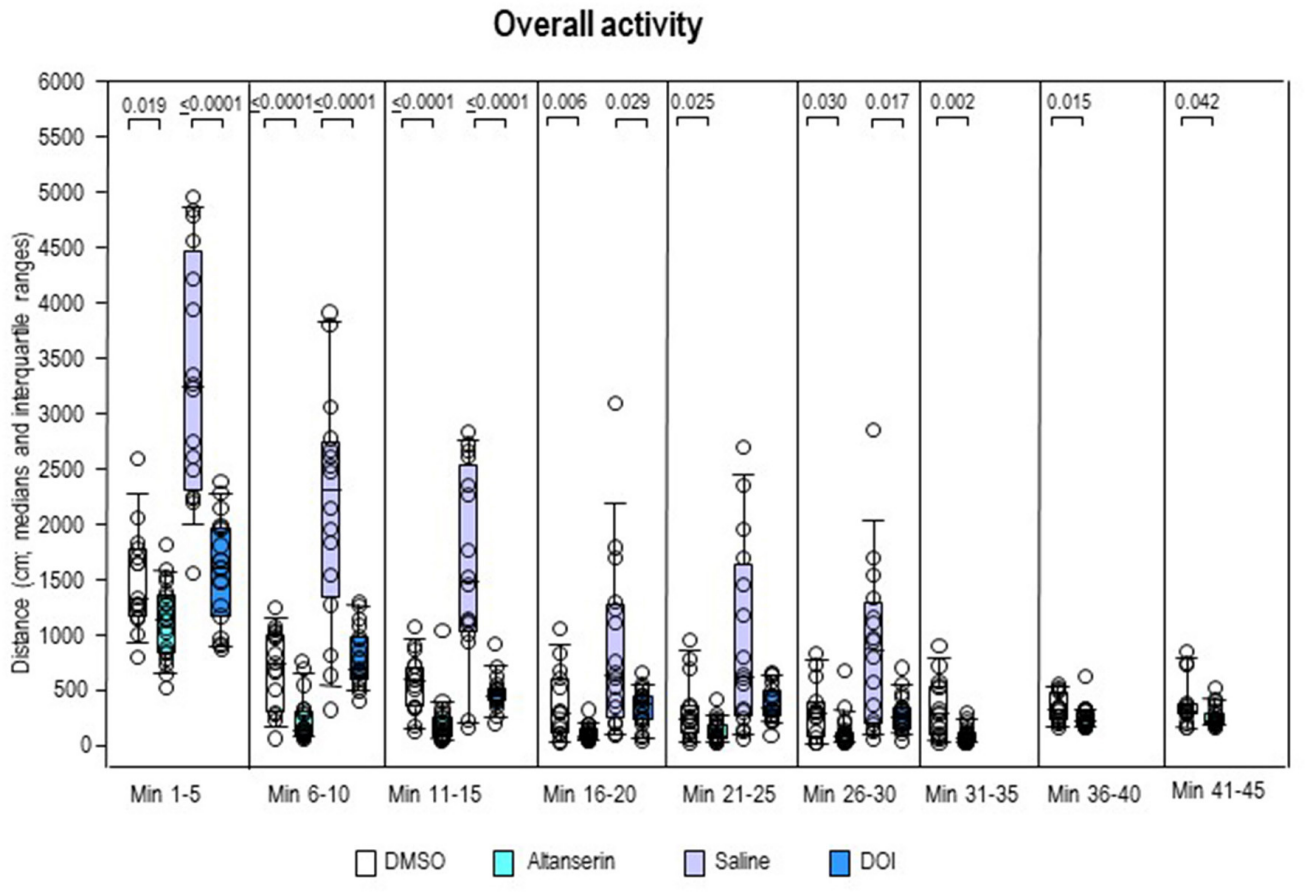
Overall activity. Traveled distance (cm) after 10 mg/kg of altanserin (turquoise), 0.5 mg/kg of 2,5-dimethoxy-4-iodoamphetamine (DOI; blue), and the respective vehicles [dimethyl sulfoxide (DMSO), white; saline, light purple]. The figure shows box and whisker plots of median overall activity in the individual 5-min bins. The 25th/75th percentiles are given in the boxes, while the 5th/95th percentiles are represented by the whiskers. The circles represent the individual animals. Groups (altanserin vs. DMSO; DOI vs. saline) were compared with the independent Mann–Whitney *U*-test (two-tailed, α = 0.05). Significant *p*-values are given.

Overall activity (Figure 6) was lower after ALT compared with DMSO in all individual 5-min bins (0.0001 ≤ *p* ≤ 0.042). After DOI, it fell short relative to SAL from min 1 to 20 (0.0001 ≤ *p* ≤ 0.029) and from min 26 to 30 (*p* = 0.017).

**Figure 7:**
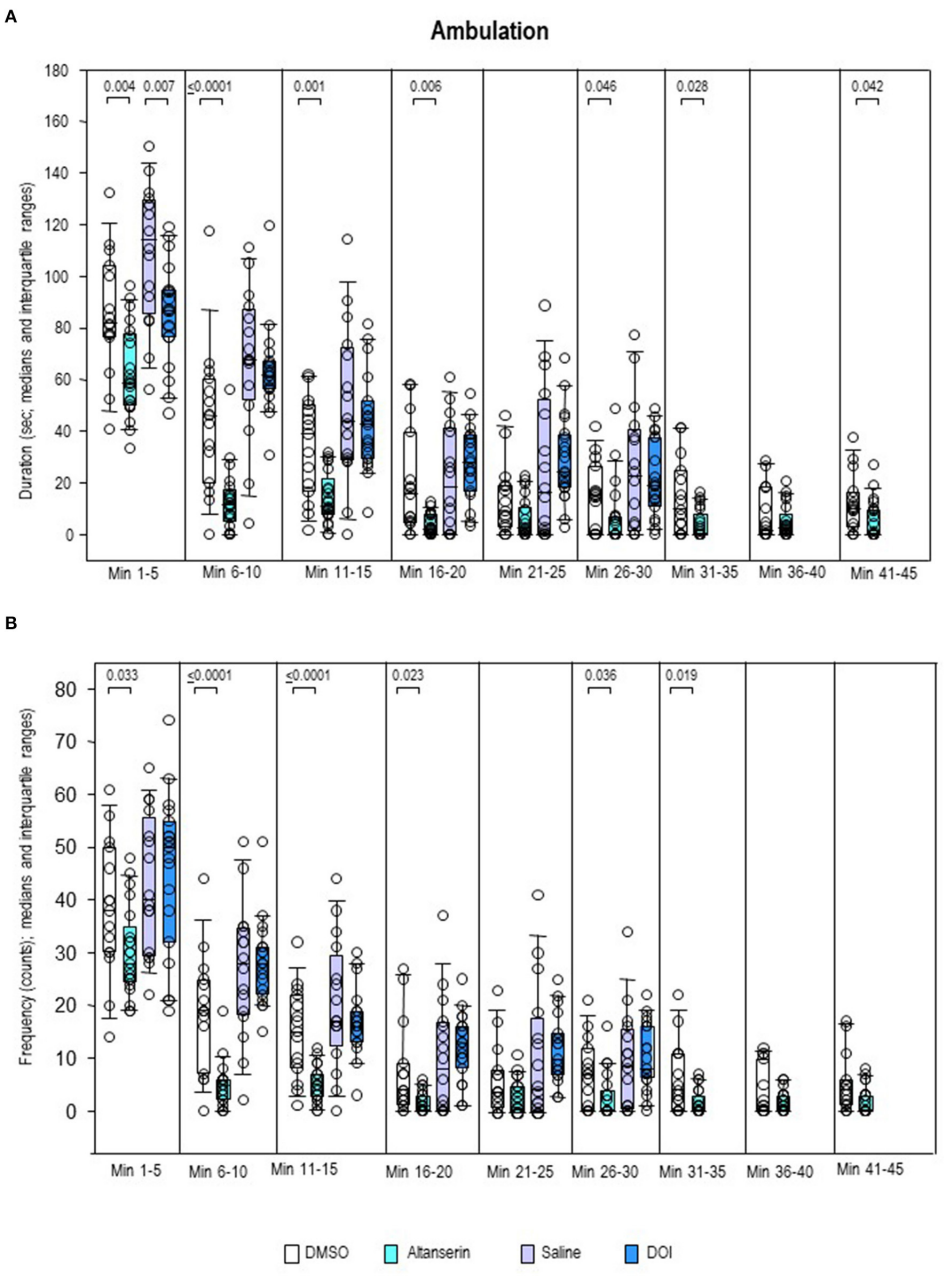
Ambulation. Duration (s) and frequency (counts) after 10 mg/kg of altanserin (turquoise), 0.5 mg/kg of 2,5-dimethoxy-4-iodoamphetamine (DOI; blue), and the respective vehicles [dimethyl sulfoxide (DMSO), white; saline, light purple]. The figure shows box and whisker plots of median ambulation durations **(A)** and frequencies **(B)** in the individual 5-min bins. The 25th/75th percentiles are given in the boxes, while the 5th/95th percentiles are represented by the whiskers. The circles represent the individual animals. Groups (altanserin vs. DMSO; DOI vs. saline) were compared with the independent Mann–Whitney *U*-test (two-tailed, α = 0.05). Significant *p*-values are given.

Inspection of the individual behavioral parameters yielded decreased duration and frequency of ambulation (Figures 7A,B) after ALT relative to DMSO from min 1 to 20 (duration, 0.0001 ≤ *p* ≤ 0.46; frequency, 0.0001 ≤ *p* ≤ 0.036). Moreover, after ALT, animals ambulated for a shorter time compared with DMSO from min 41 to 45 (*p* = 0.042). Comparisons between DOI and SAL merely yielded a decreased duration of ambulation after the former relative to the latter treatment from min 1 to 5 (*p* = 0.007).

**Figure 8:**
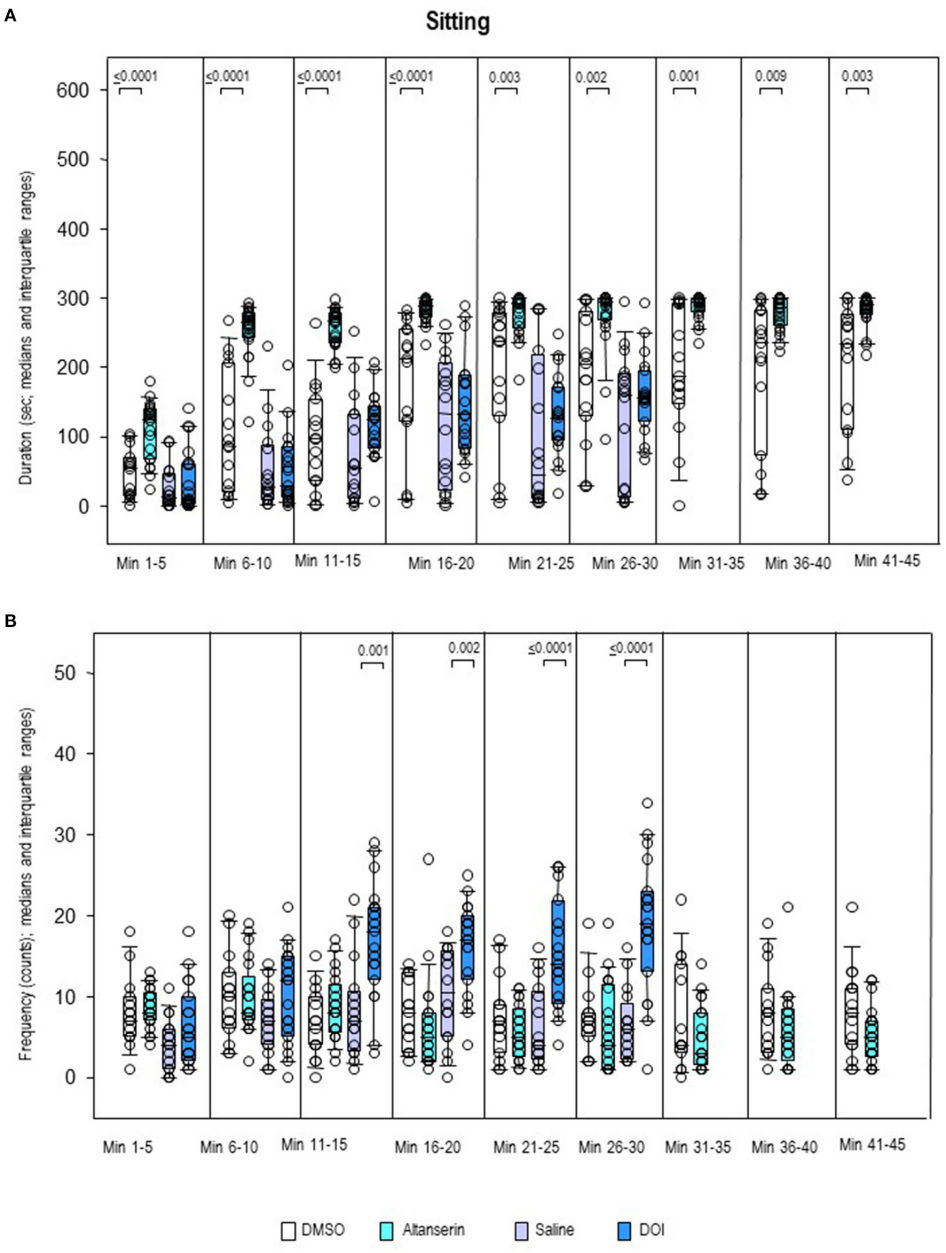
Sitting. Duration (s) and frequency (counts) after 10 mg/kg of altanserin (turquoise), 0.5 mg/kg of 2,5-dimethoxy-4-iodoamphetamine (DOI; blue), and the respective vehicles [dimethyl sulfoxide (DMSO), white; saline, light purple]. The figure shows box and whisker plots of median sitting durations **(A)** and frequencies **(B)** in the individual 5-min bins. The 25th/75th percentiles are given in the boxes, while the 5th/95th percentiles are represented by the whiskers. The circles represent the individual animals. Groups (altanserin vs. DMSO; DOI vs. saline) were compared with the independent Mann–Whitney *U*-test (two-tailed, α = 0.05). Significant *p*-values are given.

Sitting duration (Figure 8A) was increased after ALT relative to DMSO in all bins (0.0001 ≤ *p* ≤ 0.009). There were no between-group differences, however, of sitting frequency (Figure 8B). Conversely, sitting duration was unaltered after DOI compared with SAL, whereas sitting frequency was increased from min 11 to 30 (0.0001 ≤ *p* ≤ 0.002).

**Figure 9:**
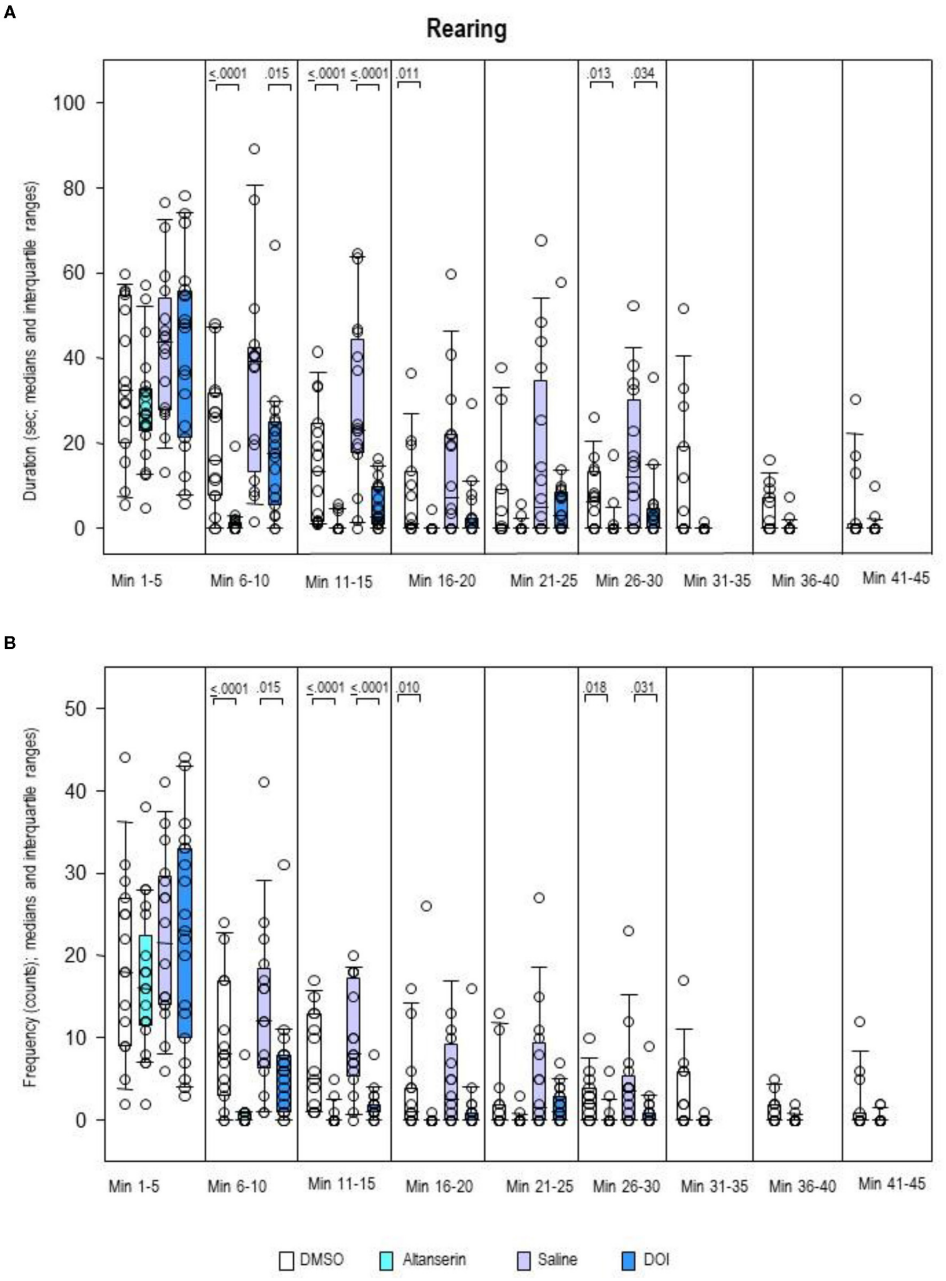
Rearing. Duration (s) and frequency (counts) after 10 mg/kg of altanserin (turquoise), 0.5 mg/kg of 2,5-dimethoxy-4-iodoamphetamine (DOI; blue), and the respective vehicles [dimethyl sulfoxide (DMSO), white; saline, light purple]. The figure shows box and whisker plots of median rearing durations **(A)** and frequencies **(B)** in the individual 5-min bins. The 25th/75th percentiles are given in the boxes, while the 5th/95th percentiles are represented by the whiskers. The circles represent the individual animals. Groups (altanserin vs. DMSO; DOI vs. saline) were compared with the independent Mann–Whitney *U*-test (two-tailed, α = 0.05). Significant *p*-values are given.

Rearing duration (Figure 9A) as well as rearing frequency (Figure 9B) was decreased after pretreatment with ALT relative to DMSO from min 6 to 20 and from min 26 to 30 (duration, 0.0001 ≤ *p* ≤ 0.013; frequency, 0.0001 ≤ *p* ≤ 0.018). After pretreatment with DOI, both rearing duration and frequency were reduced relative to SAL from min 6 to 15 and min 26 to 30 (duration, 0.0001 ≤ *p* ≤ 0.034; frequency, 0.0001 ≤ *p* ≤ 0.031).

**Figure 10:**
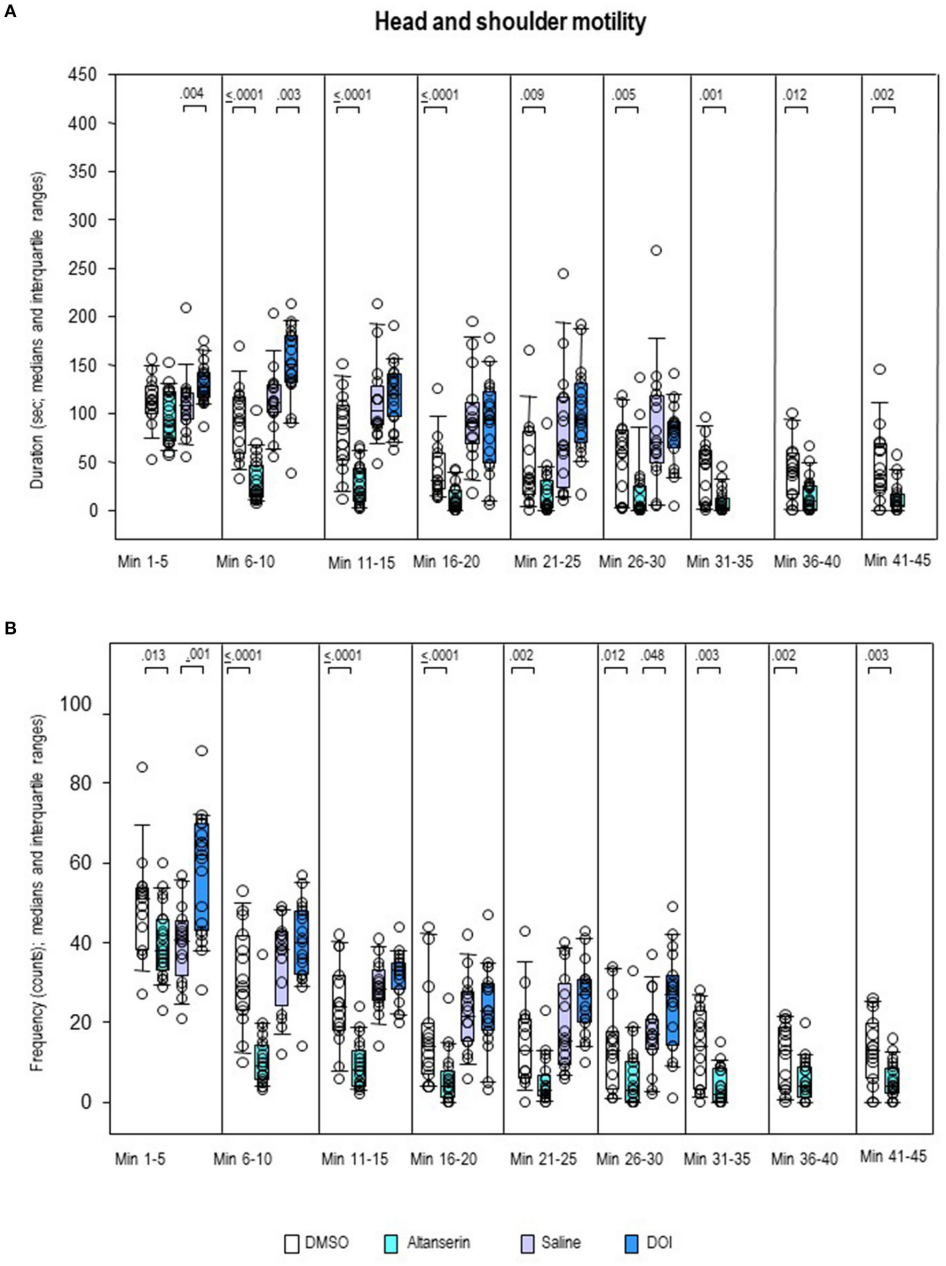
Head–shoulder motility. Duration (s) and frequency (counts) after 10 mg/kg of altanserin (turquoise), 0.5 mg/kg of 2,5-dimethoxy-4-iodoamphetamine (DOI; blue), and the respective vehicles [dimethyl sulfoxide (DMSO), white; saline, light purple]. The figure shows box and whisker plots of median durations **(A)** and frequencies **(B)** in the individual 5-min bins. The 25th/75th percentiles are given in the boxes, while the 5th/95th percentiles are represented by the whiskers. The circles represent the individual animals. Groups (altanserin vs. DMSO; DOI vs. saline) were compared with the independent Mann–Whitney *U*-test (two-tailed, α = 0.05). Significant *p*-values are given.

After ALT, the frequency of head–shoulder motility (Figure 10B) was decreased throughout the whole testing time relative to DMSO (0.0001 ≤ *p* ≤ 0.013). Except for the first 5-min bin, this also held for the duration of head–shoulder motility (Figure 10A; 0.0001 ≤ *p* ≤ 0.012). After DOI, the frequency of head–shoulder motility was elevated relative to SAL from min 1 to 5 (*p* = 0.001) and min 26 to 30 (*p* = 0.048). An increase of duration of head–shoulder motility was observed from min 1 to 10 (*p* = 0.004 and 0.003, respectively).

## Discussion

### D_2/3_R Binding

The present study presents the first *in vivo* imaging evidence on the effects of 5-HT_2A_R antagonistic and agonistic challenges on subcortical and neocortical DA functions: systemic treatment with the 5-HT_2A_R antagonist ALT in a dose of 10 mg/kg significantly increased D_2/3_R binding in vHIPP (+22%) relative to vehicle, while systemic treatment with the 5-HT_2A_R agonist DOI in a dose of 0.5 mg/kg significantly augmented D_2/3_R binding CP (+17%), FC (+16%), MC (+29%), and vHIPP (+30%).

In previous studies on rats, systemic l-DOPA as well as GABA_A_R agonist and *N*-methyl-d-aspartate (NMDA)R antagonist treatment reduced [^123^]IBZM binding, whereas GABA_A_R antagonist and NMDAR agonist treatment elevated [^123^]IBZM binding to the neostriatal D_2/3_R (Nikolaus et al., 2016, 2018, 2019). Since [^123^]IBZM competes with endogenous DA molecules for D_2/3_R binding sites, the observed decreases and increases of D_2/3_R binding may be interpreted to reflect increases and decreases, respectively, of synaptic DA (Laruelle, 2000). Hence, it can be assumed that, also in the present study, the regional elevations of D_2/3_R binding elicited by ALT and DOI were due to decreased DA concentrations in these areas.

Under the present experimental conditions, we observed a significant elevation of D_2/3_R binding (indicative of decreased DA levels) in the vHIPP after 10 mg/kg of ALT. However, in contrast to the results obtained by Dewey et al. (1995) on baboons and rats after 1 and 20 mg/kg of ALT using PET and *in vivo* microdialysis, respectively, no significant decreases of neostriatal D_2/3_R binding were detected. This may be accounted for by the differences of method: (1) non-invasive *in vivo* imaging of rats vs. invasive *in vivo* microdialysis of rats in the study of Dewey et al. (1995), (2) anesthetized rats vs. freely moving rats in the study of Dewey et al. (1995), (3) adult rats (mean weight, 466 g) vs. adolescent rats (200–300 g) in the study of Dewey et al. (1995), and, finally, (4) *in vivo* imaging of rats vs. *in vivo* imaging of baboons in the study of Dewey et al. (1995). Another likely reason might be the dosage, since Dewey et al. (1995) applied either a tenth of the dose or twice the dose (1 or 20 mg/kg, respectively) administered in our investigation. In order to shed further light on the impact of dosage, a dose–response curve should be established by future imaging or *in vivo* microdialysis studies after increasing doses of ALT.

The present findings showed significant increases of D_2/3_R binding (indicative of decreased DA levels) after systemic DOI in CP, FC, MC, and vHIPP. Since the affinity of DOI for the 5-HT_2A_R binding site exceeds its affinity for the 5-HT_2C_R by one order of magnitude (Canal et al., 2013), and HT_2C_R is also related to DA efflux (Alex et al., 2005), it cannot be entirely excluded that the effects of DOI may have also partially been induced by 5-HT_2C_R action. The present result of decreased DA in the CP agrees with the outcome of a previous *in vivo* microdialysis study, reporting a reduction of striatal DA efflux upon infusion of DOI (10 and 20 μM) into this region (Ng et al., 1999). Apart from the lack of a significant alteration of D_2/3_R binding in the NAC (after DOI in a dose of 2 mg/kg i.p.), which was also reported by Ichikawa and Meltzer (1995), our findings on D_2/3_R binding, however, are not consistent with the precedent microdialysis findings after systemic application of DOI (Gudelsky et al., 1994; Ichikawa and Meltzer, 1995; Gobert and Millan, 1999), which reported either no effect on DA levels in the CP (2 mg/kg i.p., Gudelsky et al., 1994; 2.5 mg/kg i.p., Ichikawa and Meltzer, 1995) or increased DA levels in the FC (0.1–10 mg/kg s.c., Gobert and Millan, 1999). The dose of 0.5 mg/kg of DOI lies in the range of doses (0.1–10 mg/kg) applied in the other investigations (Gudelsky et al., 1994; Ichikawa and Meltzer, 1995; Gobert and Millan, 1999), which precludes dosage as a relevant factor for the inconsistency of outcomes. However, a likely reason for the observed discrepancy, also here, are the methodological differences: we employed a non-invasive *in vivo* imaging approach in contrast to the invasive microdialysis studies of Gudelsky et al. (1994), Ichikawa and Meltzer (1995), and Gobert and Millan (1999). Moreover, we used adult rats in contrast to the adolescent animals employed in the other investigations (Gudelsky et al., 1994, 225–300 g; Ichikawa and Meltzer, 1995, 200–300 g; Gobert and Millan, 1999, 200–220 g).

### 5-HT_2A_R Agonistic and Antagonistic Actions Within Cerebral Networks

After 5-HTergic challenges as well as vehicles, network analyses of regional D_2/3_R BPs indicated distinct relations between the individual brain regions. Thereby, irrespective of the applied treatment, the same framework of functional connections between NAC and CP, NAC and THAL, CP and THAL, SN/VTA and THAL, SN/VTA and vHIPP, and FC and MC was obtained. Interestingly, however, after SAL, a strong positive connection was found between NAC and THAL, which became negative after treatment with DOI. This also held for the connections between NAC and PC, SN/VTA and FC, and SN/VTA and MC. Furthermore, strong positive connections between NAC and FC, CP and SN/VTA, CP and THAL, CP and PC, CP and dHIPP, SN/VTA and dHIPP, THAL and vHIPP, PC and dHIPP, FC and PC, and MC and vHIPP and strong negative connections between THAL and PC as well as dHIPP and vHIPP were added.

In the mammalian brain, 5-HTergic fibers extend to the SN and VTA (Oades and Halliday, 1987; Wirtshafter et al., 1987), which both express high amounts of 5-HT_2A_R binding sites (Doherty and Pickel, 2000; López-Giménez et al., 2001). 5-HT_2A_R action is known to increase the efflux of both GABA (Jiang et al., 2009) and GLU in the central nervous system (Meller et al., 2002). Previous studies with SB 242084 and Ro 60-0175 as receptor antagonist and agonist, respectively, additionally, have shown that the 5-HT_2_R subtype decreased DA release in the CP by increasing GABAergic inhibition in the SN (Burke et al., 2014). Hence, it may be hypothesized that, after treatment with DOI, increased GABAergic inhibition outweighed GLUergic excitation in the sites of origin of DAergic fibers, leading to a reduction of DA efflux in the neostriatal target region as reflected by the elevation of radioligand binding to the striatal D_2/3_R. The 5-HT_2A_R agonistic action exerted on the CP *via* the SN, moreover, is indicated by the strong positive connection between both regions obtained in network analyses. The CP is inhibited by GABAergic microcircuits (Groves, 1983). Since it also receives ascending 5-HTergic fibers from the dorsal raphe nucleus (Steinbusch et al., 1980), it can be assumed that DOI, furthermore, facilitated the GABAergic inhibition exerted *via* these microcircuits, adding to the decrease of available DA. In the DAergic system, DA concentrations are regulated by autoreceptors of the D_2_R subtype localized at the presynaptic terminal (Langer, 1974). Remarkably, the DOI-induced reduction of available DA appeared to be so high that the inhibitory feedback mechanism of D_2_ autoreceptors was not effective in normalizing synaptic DA levels in the CP, at least in the present time window between DOI administration and D_2/3_R imaging at 75 min post-challenge.

The THAL (Moore et al., 1978) and neocortex also receive 5-HTergic projections (Kievit and Kuypers, 1975). Both regions exhibit high densities of GABA (Neto et al., 2006; Mann et al., 2009) and GLU binding sites (Salt et al., 1996; Sherman, 2014). Possibly, the 5-HT_2A_R agonistic treatment increased GABAergic input from the THAL to the neocortex relative to GLUergic input, resulting in a net reduction of available DA in the FC and MC as reflected by the observed elevation of D_2/3_R binding in these regions. In turn, it may be assumed that decreased DAergic input from the CP to the neocortex together with the increased availability of GABA in the latter region incurred a net reduction of GLUergic input to the THAL, resulting in normal DA levels, as observed in the present investigation. After both SAL and DOI, network analyses yielded strong positive connections between FC and MC as well as THAL and FC and a strong negative connection between SN/VTA and MC. Interestingly, however, after DOI, the positive connections between NAC and PC, SN/VTA and FC, and SN/VTA and MC turned negative, while a negative connection between THAL and PC and two positive connections between NAC and both FC and MC were introduced. This implies an involvement of mesolimbocortical as well corticocortical projections in the regulation of synaptic DA. Future *in vivo* imaging studies with different doses of DOI are required in order to further elucidate this matter.

The HIPP receives DAergic neurons originating in the VTA (Nazari-Serenjeh et al., 2011), which—along with its most prominent projection area, the NAC—displayed no changes of D_2/3_R binding under the present experimental conditions. Previously, DOI was found to elevate GLU levels in the VTA (Pehek et al., 2006). Given the known facilitatory effect of 5-HT_2A_Rs also on GABAergic neurotransmission (Jiang et al., 2009), it may be that the elevation of GABAergic inhibition exerted by DOI in the VTA, in analogy to its actions in the SN, outweighed GLUergic excitation, leading to a reduction of DA levels in the hippocampal target regions of ventral tegmental projections, which is reflected by the observed elevation of radioligand binding to the D_2/3_R in the vHIPP. The contribution of SN/VTA is underlined by the strong positive connection between this region and vHIPP obtained in network analysis. Interestingly, however, DOI also introduced strong positive connections between THAL and dHIPP, THAL and vHIPP, and PC and dHIPP as well as a strong negative connection between dHIPP and vHIPP. The emergence of limbic connections after DOI relative to SAL reflects the relevance of the 5-HTergic system for limbic function. Also here, further investigations are required in order to gain more insight into the 5-HT-triggered regulatory mechanisms of limbic DA.

After DMSO, strong positive connections were found between NAC and THAL and FC and PC, which turned negative after ALT. Conversely, a strong negative connection between NAC and PC turned positive after ALT. Besides, ALT introduced strong positive connections between NAC and vHIPP, SN/VTA and MC, THAL and PC, THAL and dHIPP, MC and PC, MC and dHIPP, and MC and vHIPP as well as strong negative connections between NAC and dHIPP and PC and vHIPP.

Since both GABA efflux (Jiang et al., 2009) and GLU efflux (Meller et al., 2002) are stimulated by 5-HT_2A_R action, application of a 5-HT_2A_R antagonist likely reduces the inhibitory GABAergic as well as the excitatory GLUergic effects of 5-HT. Hence, the increases of DA efflux in the CP after application of ALT reported by Dewey et al. (1995) may have been due to a decrease of GABAergic inhibition relative to the reduction of GLUergic excitation, leading to a net increase of available DA. Presumably, in the present study, a decrease of GABAergic inhibition induced by the dose of 10 mg/kg of ALT was either too short-termed or so low that it could be compensated by the reduction of GLUergic excitation, incurring no alteration of D_2/3_R binding in the CP indicative of altered synaptic DA levels. This presumption is underlined by the lack of connection between the SN/VTA and CP in network analyses.

It may be assumed that, in contrast to DOI, the 5-HT_2A_R antagonist also did not decrease the GABAergic input from the THAL to the neocortex relative to the GLUergic input, resulting in unaltered DA levels in the cortical regions. This is supported by the lack of connection between THAL and FC in network analysis.

For the vHIPP, it can be hypothesized that, in a reversal of the actions exerted by DOI, a reduction of GLUergic excitation outweighed the simultaneous decrease of GABAergic inhibition, also—ultimately—resulting in a decline of DA levels (and the observed increase of D_2/3_R binding) in the vHIPP. Also here, the contribution of SN/VTA is underlined by the strengthened positive connection between this region and vHIPP obtained in network analysis.

Also, ALT introduced strong connections between regions of the mesolimbothalamocortical system including positive associations between NAC and PC, NAC and vHIPP, SN/VTA and dHIPP, THAL and dHIPP, and THAL and PC and negative associations between NAC and THAL, NAC and dHIPP, and MC and vHIPP. Thereby, however, the 5-HT_2A_R agonist in contrast to the 5-HT_2A_R antagonist induced strong positive connections between CP and PC, SN/VTA and CP, SN/VTA and dHIPP, THAL and FC, and PC and dHIPP and a strong negative connection between NAC and PC, and dHIPP and vHIPP. Moreover, it strengthened the positive connections between the THAL and both parts of the HIPP and the negative connection between SN/VTA and FC. This implies that DOI exerted more varied and stronger effects in comparison to ALT, which is also reflected by the fact that DOI affected D_2/3_R binding also in the striatal and neocortical regions.

### Behavior

The 5-HT_2A_R agonist as well as antagonist decreased parameters of motor activity (overall activity and ambulation duration) and active exploratory behavior (rearing duration and frequency). Thereby, after DOI, reductions of motor activity were confined to min 1–20 (overall activity) and min 1–5 (ambulation duration). However, while DOI increased duration and frequency of explorative head–shoulder movements, but did not affect passive immobility (sitting duration), ALT decreased explorative head–shoulder motility and increased passive immobility. In sum, the reduction of motor activity was more pronounced after ALT, while the reductions of active (vertical) exploration were comparable after ALT and DOI.

The finding of diminished ambulatory behavior after ALT confirms the results obtained in a previous study (Kennett, 1992). Likewise, results obtained after DOI corroborate precedent findings of reduced ambulatory (Hillegaart et al., 1996; Krebs-Thomson et al., 1998; Zaniewska et al., 2009) and rearing behaviors (Hillegaart et al., 1996; Krebs-Thomson et al., 1998; Hawkins et al., 2002) upon systemic pretreatment with DOI. They are not consistent, however, with the findings on ambulation by Hawkins et al. (2002), who observed no effect on motor activity after i.c.v. administration of 0.02–0.2 mg as well as s.c. administration of 0.1–1 mg/kg. A likely reason for this discrepancy is that Hawkins et al. (2002) performed their open field test 10 min after a tail-pinch stress test (Hawkins et al., 2002), which may have influenced the animals' activity.

Joint investigations of motor/exploratory behaviors and D_2/3_R imaging have shown that, in mature rats, decreases of regional D_2/3_R binding (indicative of elevated DA) were related to decreases of motor/exploratory activity (e.g., Nikolaus et al., 2016, 2018, 2019). Also, in the present study, parameters of motor activity and vertical exploratory behavior were reduced by 5-HT_2A_R agonistic as well as antagonistic treatment, with decreases, however, more pronounced and extended over a longer period after ALT compared with DOI. This may reflect the rise of extracellular DA, which was reported for either compound in *in vivo* microdialysis studies shortly post-injection (Dewey et al., 1995; Gobert and Millan, 1999). In context with our previous findings, the reduction of motor/explorative behaviors is in striking contrast to the reductions of D_2/3_R binding observed in vHIPP after ALT and in CP, FC, MC, and vHIPP after DOI, which, rather, indicate decreased DA levels in these regions. This infers that the regional elevations of synaptic DA indicated by the behavioral findings could not be visualized in the chosen time windows for *in vivo* imaging (90–150 min post-challenge for ALT and 75–135 min post-challenge for DOI). After ALT and DOI, DA levels peaked from 40 to 60 min (Dewey et al., 1995) and 20 to 60 min post-challenge (Gobert and Millan, 1999), respectively, with a steady reduction thereafter. Apparently, in the time between radioligand application and the achievement of binding equilibrium, the decline of DA levels incurred a decrease of competition for D_2/3_R binding sites, which, in conjunction with the higher affinity of [^123^I]IBZM (K_D_ = 0.3 nM; Verhoeff et al., 1991) relative to the endogenous ligand (K_D_ = 1.2 nM, de Paulis et al., 1988) resulted in the observed reductions of D_2/3_R binding. Future *in vivo* imaging studies after various concentrations of ALT and DOI and with radioligand injection at various times post-challenge are required to further elucidate the time dependency of the effects, which are exerted by ALT and DOI on regional D_2/3_R binding (and DA levels).

### Appraisal

For one, also the vehicle DMSO might have induced pharmacological effects. Unpublished results have shown that neostriatal D_2/3_R binding after DMSO was elevated relative to SAL. Also in the present study, tentative comparisons of D_2/3_R BPs have yielded increased receptor binding in NAC, CP, FC, and MC of DMSO-treated relative to SAL-treated rats. Moreover, there is evidence that DMSO decreased motor/exploratory behaviors relative to SAL. In the present study, such differences were observed for overall activity (min 1–30), ambulation duration (min 1–10), sitting duration (min 1–5 and 26–30), sitting frequency (min 1–5), rearing duration (min 11–15), duration of head–shoulder motility (min 16–20), and frequency of head–shoulder motility (min 6–10). Hence, it cannot be excluded that the effects of ALT added upon pharmacological effects of DMSO. For this reason, behaviors as well as regional BPs were only compared between 5-HTergic challenges and the respective vehicle but not between ALT and DOI.

Second, in the present *in vivo* studies, the maximum VOI diameters were either in the range of or beyond the spatial resolution of the employed imaging tool. It must be considered, however, that in those portions of VOIs, whose diameters are smaller than the FWHM, the exact quantification of D_2/3_R binding may be hampered by partial volume effects leading to underestimations of radioligand accumulation. A further source of error may be spillover from regions with high radioligand accumulation such as the extraorbital Harderian glands to the adjacent VOIs of the FC, CP, and NAC, or from the CP to NAC, THAL, and dHIPP, causing overestimations of radioligand binding. However, since this pertains to SPECT measurements both in baseline and after challenge, the exactitude of (semi)quantitative values in either condition, but not the comparability of data between baseline and challenge, may have been biased.

Third, *in vivo* imaging findings may have been flawed by the employment of the NMDAR antagonist ketamine as anesthetic. Since ketamine has previously been shown to enhance DA release and reduce D_2/3_R binding in rats (e.g., Tsukada et al., 2000), it cannot be dismissed that also in the present study increased DA levels due to ketamine usage reduced the amounts of visible regional D_2/3_R binding after both ALT and DOI. However, effects on neostriatal and/or ventrostriatal DA are exerted by practically all known anesthetics, including pentobarbital, propofol, halothane, chloral hydrate, and isoflurane (for review, see Müller et al., 2011). Since ketamine was employed in all our previous *in vivo* imaging studies, we, therefore, decided to use it also in the present investigation. As this possible pitfall concerns the outcome of SPECT measurements after 5-HTergic challenges as well as after treatment with the respective vehicles, the obtained BPs remain comparable between conditions. The publications of Gudelsky et al. (1994) and Gobert and Millan (1999) do not give information on the state of the animals during microdialytic sampling. However, in the experiment of Ichikawa and Meltzer (1995), who observed no changes of striatal DA concentrations upon infusion of DOI, dialysates were drawn, while the rats were freely moving. Hence, it must be taken into account that anesthesia as such may have contributed to the differences between studies.

Fourth, in this and other investigations (e.g., Nikolaus et al., 2016, 2017, 2018, 2019), effects of DAergic, GABAergic, glutamatergic, and 5-HTergic challenges on regional D_2_R binding and motor/exploratory behaviors have merely been assessed in male rats. The main reason for not using female animals is their estrous cycle, which is characterized by four phases with distinct fluctuations of estrogen and progesterone concentrations (Butcher et al., 1974). Thus, if the hormonal state of the female rodent is to be accounted for, the number of subjects per experiment multiplies, since four times more females than males have to be examined. Nevertheless, there is not only evidence of sex differences in incidence and onset of stress-related and other psychiatric disorders (for review, see ter Horst et al., 2012) but also of an interaction between ovarian hormone and monoamine function (Janowsky et al., 1971). As a consequence, future studies should also be conducted on female rats in order to duly assess the impact of the hormonal state on D_2/3_R binding under a given pharmacological challenge.

## Conclusion

Taken together, the 5-HT_2A_R agonistic DOI increased D_2/3_R binding (and presumably decreased DA) in the CP as well as in the limbic and neocortical target regions (vHIPP, FC, and MC) of ascending 5-HTergic as well as DAergic projections. After application of the 5-HT_2A_R antagonistic ALT, the increase of D_2/3_R binding (and the decrease of DA) was confined to vHIPP. Both ALT and DOI decreased parameters of motor activity and active (vertical) exploration. However, while DOI increased explorative head–shoulder motility, ALT decreased explorative head–shoulder motility and increased passive immobility. The reductions of motor/exploratory activities contrast the regional reductions of D_2/3_R binding, as they indicate elevated DA levels at the time of behavioral measurements. It may be concluded that ALT and DOI modulated DA in the individual regions of the nigrostriatal and mesolimbocortical pathways differentially and in a time-dependent fashion.

## Data Availability Statement

The raw data supporting the conclusions of this article will be made available by the authors, without undue reservation.

## Ethics Statement

The animal study was reviewed and approved by Landesamt für Natur, Umwelt und Verbraucherschutz Nordrhein-Westfalen, Recklinghausen, Germany.

## Author Contributions

SN, JPH, GA, and H-WM: experimental design. SN, H-JW, YM, and CD: performance of imaging and behavioral studies. SN and MB: evaluation and statistical analysis. SN, JPH, and H-WM: interpretation of findings. SN, H-JW, MB, CA, EM, YM, HH, CD, JPH, and H-WM: writing and editing of the manuscript. All authors contributed to the article and approved the submitted version.

## Conflict of Interest

The authors declare that the research was conducted in the absence of any commercial or financial relationships that could be construed as a potential conflict of interest.

## Publisher's Note

All claims expressed in this article are solely those of the authors and do not necessarily represent those of their affiliated organizations, or those of the publisher, the editors and the reviewers. Any product that may be evaluated in this article, or claim that may be made by its manufacturer, is not guaranteed or endorsed by the publisher.
